# Association of the Modified Endothelial Activation and Stress Index with MASLD and Liver Fibrosis: Effect Modification by Magnesium Intake

**DOI:** 10.5152/tjg.2026.25591

**Published:** 2026-04-21

**Authors:** Xin Li, Zizheng Huang

**Affiliations:** 1 Chongqing Medical University Medical School of Artificial Intelligence, Chongqing, China; 2 National Local Joint Engineering Research Center for Precision Surgery & Regenerative Medicine, Shaanxi Provincial Center for Regenerative Medicine and Surgical Engineering, The First Affiliated Hospital of Xi’an Jiaotong University, Shaanxi, China

**Keywords:** Liver fibrosis, magnesium, metabolic dysfunction–associated steatotic liver disease, modified endothelial activation and stress index, NHANES

## Abstract

**Background/Aims:**

Few studies have reported the effect of magnesium status on the endothelial dysfunction–related risk for the development of MASLD and liver fibrosis. The current study aimed to explore the association of modified endothelial activation and stress index (mEASIX) with metabolic dysfunction–associated steatotic liver disease (MASLD) and liver fibrosis and to examine the effect of magnesium intake on this association.

**Materials and Methods:**

A cross-sectional study was conducted using data from 5960 participants in the National Health and Nutrition Examination Survey 2017-2020. The mEASIX was calculated based on lactate dehydrogenase levels, high-sensitivity C-reactive protein levels, and platelet counts. Metabolic dysfunction–associated steatotic liver disease was defined as the presence of hepatic steatosis along with at least one of the 5 cardiometabolic risk factors. Liver fibrosis was defined as a liver stiffness measurement ≥ 8.0 kPa. Magnesium (Mg) intake was assessed using the 24-hour dietary recall data. Multivariate logistic regression analyses were performed to examine the associations of mEASIX with MASLD and liver fibrosis.

**Results:**

The mEASIX was positively associated with MASLD and liver fibrosis (MASLD: odds ratio [OR] in Q2 = 1.62, Q3 = 2.38, and Q4 = 2.70; liver fibrosis: OR in Q3 = 1.84 and Q4 = 2.73; all *P* < .05). The mEASIX revealed nonlinear relationships with MASLD and liver fibrosis. A significant interaction between magnesium intake and mEASIX was observed in relation to MASLD. Stratified analyses further demonstrated that the association between high mEASIX (≥1.2) and the risk of MASLD and liver fibrosis was more pronounced among participants with low magnesium intake compared to those with high magnesium intake.

**Conclusion:**

The current study demonstrated positive nonlinear associations of mEASIX with MASLD and liver fibrosis. Low magnesium intake may exacerbate this endothelial dysfunction–related risk for MASLD and liver fibrosis.

**Main Points**

- There were positive associations of modified endothelial activation and stress index (mEASIX) with metabolic dysfunction–associated steatotic liver disease (MASLD) and liver fibrosis.
- Associations of mEASIX with MASLD and liver fibrosis were nonlinear.
- Magnesium intake changed the association between mEASIX and MASLD/liver fibrosis.

## Introduction

Over the past 3 decades, the global burden of metabolic dysfunction–associated steatotic liver disease (MASLD) and MASLD-related fibrosis/cirrhosis has increased.^1^^-^^3^ The presence of these chronic liver diseases further leads to a substantial increase in liver cancer, cardiovascular disease, and mortality. Early identification of high-risk populations for MASLD/liver fibrosis and finding potential modifiable factors are vital for the prevention, control and reduction of heavy burden due to liver diseases.

Endothelial cell dysfunction appears to play a key role in the development of MASLD and liver fibrosis via numerous mechanisms, including the regulation of inflammatory processes and immune responses.^4^^,^^5^ However, evidence from large-scale population studies remains insufficient, primarily because of the complexity and high cost of the detection methods. The endothelial activation and stress index (EASIX), a score calculated using routine clinical markers including lactate dehydrogenase, platelet counts, and creatinine, was initially proposed to predict the survival of patients undergoing allogeneic stem cell transplantation.^6^^,^^7^ Recent evidence has shown that EASIX is associated with the prognosis of acute-on-chronic liver failure,^8^ cardiometabolic diseases,^9^^-^^14^ and various cancers.^15^^-^^18^ However, it remains unclear whether EASIX is related to MASLD and liver fibrosis. Additionally, the modified EASIX (mEASIX), in which creatinine is replaced with C-reactive protein, has been shown to be a better predictor of survival compared to EASIX in patients with Hodgkin’s lymphoma.^19^ Considering the significance of inflammation, it was hypothesized that mEASIX was associated with the development of MASLD and liver fibrosis.

In addition, as one of the essential minerals, magnesium plays a key role in improving oxidative stress and inflammation.^20^^,^^21^ Nevertheless, a few studies have reported the effect of magnesium status on the endothelial dysfunction–related risk for the development of MASLD and liver fibrosis. Evidence showed that endothelial cells cultured in low magnesium rapidly activate the nuclear factor-kappa B (NF-κB) pathway, which correlates with marked alterations of the cytokine network.^22^^,^^23^ These upregulated inflammatory cytokines play a key role in the pathogenesis of MASLD/liver fibrosis involving endothelial cells dysfunction.^4^^,^^24^ Thus, it is hypothesized that magnesium can affect the risk of endothelial function–related MASLD/liver fibrosis by altering inflammation pathways.

Therefore, a cross-sectional study is conducted to explore the associations of mEASIX with MASLD and liver fibrosis and to examine the effect of magnesium intake on this association.

## Materials and Methods

### Study Design and Population

This was a cross-sectional study that used publicly available data from the National Health and Nutrition Examination Survey (NHANES). The NHANES was approved by the National Center for Health Statistics (NCHS) Research Ethics Review Board. Approval of the NCHS Research Ethics Review Board is available on the website https://www.cdc.gov/nchs/nhanes/about/erb.html. All participants provided written informed consent. All NHANES data from the NCHS were de-identified and kept anonymous; thus, secondary analyses of these data did not require additional ethical approval or informed consent. Access to the data was made on July 15, 2025.

**Figure 1.**
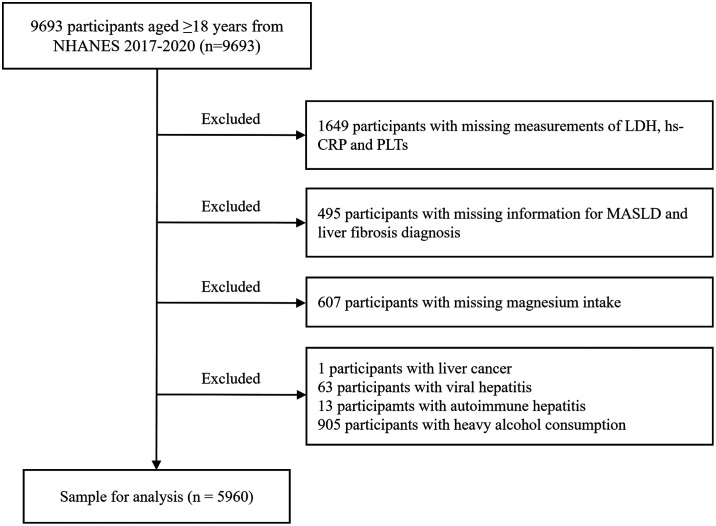
Flowchart of study population selection.

A total of 9693 participants aged 18 years and older were initially included from the NHANES 2017-2020. Participants were excluded if they met the following criteria: (1) incomplete data on lactate dehydrogenase (LDH), high-sensitivity C-reactive protein (hs-CRP) and platelet counts (PLTs) (n = 1649); (2) incomplete information for MASLD or liver fibrosis diagnosis (n = 495); (3) missing magnesium intake (n = 607); (4) had liver cancer (n = 1); (5) hepatic steatosis caused by other reasons, including viral hepatitis (n = 63), autoimmune hepatitis (n = 13), and heavy alcohol consumption (n = 905). Finally, 5960 participants were included in the analysis (Figure 1).

### Modified Endothelial Activation and Stress Index

**Modified endothelial activation and stress index** = LDH [U/L] × hs-CRP [mg/dL] / PLTs [10^9^/L].^19,25^ The mEASIX was grouped by quartiles (Q1: ≤ 0.49, Q2: 0.49-1.09, Q3: 1.09-2.51, Q4: > 2.51) and divided into 2 groups according to the restricted cubic spline (RCS) cutoff (high mEASIX ≥ 1.2, low mEASIX < 1.2).

Lactate dehydrogenase levels were measured by a Roche Cobas 6000 (c501 module) analyzer. The PLTs were determined using a Coulter DxH 800 analyzer. High-sensitivity C-reactive protein (hs-CRP) levels were measured using a 2-reagent, immunoturbidimetric system. Details of the laboratory procedures and quality assurance can be accessed in the following links.

Lactate dehydrogenase: https://wwwn.cdc.gov/Nchs/Data/Nhanes/Public/2017/DataFiles/P_BIOPRO.htm#

Platelet counts: https://wwwn.cdc.gov/Nchs/Data/Nhanes/Public/2017/DataFiles/P_CBC.htm

High-sensitivity C-reactive protein: https://wwwn.cdc.gov/Nchs/Data/Nhanes/Public/2017/DataFiles/P_HSCRP.htm

### Metabolic Dysfunction–Associated Steatotic Liver Disease and Liver Fibrosis

Metabolic dysfunction–associated steatotic liver disease was defined as the coexistence of hepatic steatosis (controlled attenuation parameter ≥ 274 dB/m)^26^ and at least one of the following 5 cardiometabolic risk factors: (1) body mass index ≥ 25 kg/m^2^ or waist circumference > 94 cm (men)/80 cm (women), (2) glycated hemoglobin ≥ 5.7% or fasting glucose ≥ 100 mg/dL or diabetes or use of antidiabetic agents, (3) blood pressure ≥ 130/85 mmHg or use of antihypertensive agents, (4) fasting triglyceride ≥ 150 mg/dL or use of lipid-lowering drugs, (5) plasma high-density lipoprotein cholesterol < 40 mg/dL (men) and < 50 mg/dL (women) or use of lipid-lowering drugs.^27^ Liver fibrosis was defined as a median liver stiffness ≥ 8.0 kPa.^26^^,^^27^

The controlled attenuation parameter and median liver stiffness were obtained using FibroScan (model 502 V2 Touch) through ultrasound and vibration controlled transient elastography technology, which has been validated accurately. A detailed description of quality assurance can be found in the procedure manual (https://wwwn.cdc.gov/Nchs/Data/Nhanes/Public/2017/DataFiles/P_LUX.htm).

### Magnesium Intake

The magnesium intake was assessed using the 24-hour dietary recall data, including dietary source and the supplement. In the current study, it was grouped as high Mg intake group and low Mg intake group according to the median (286.74 mg/day). The average magnesium intake from 2 non-consecutive days was used for sensitivity analysis. The second dietary recall was collected by telephone 3-10 days later after the first in-person dietary recall. The median of the average magnesium intake for 2 nonconsecutive days was 287.95 mg/d.

### Covariables

Covariables used in the current study were as follows: Demographic factors included age, gender, race, education level, and poverty income ratio (PIR). Lifestyle factors included smoking, drinking, physical activity, and total energy intake. Laboratory parameters included aspartate aminotransferase (AST), alanine aminotransferase (ALT), gamma glutamyl transferase (GGT), and albumin. Comorbidities included overweight (body mass index ≥ 25 kg/m^2^), diabetes (glycated hemoglobin ≥ 6.5%, fasting glucose ≥ 126 mg/dL, self-report, or use of antidiabetic agents), hypertension (blood pressure ≥ 140/90 mmHg, self-report, or use of antihypertensive agents), dyslipidemia (triglyceride ≥ 150 mg/dL, total cholesterol ≥ 200 mg/dL, high-density lipoprotein cholesterol ≤ 40 mg/dL, low-density lipoprotein cholesterol ≥ 130 mg/dL, self-report, or use of lipid-lowering drugs), cardiovascular diseases (CVDs) (self-report of angina, coronary heart disease, myocardial infarction, congestive heart failure, or stroke), and chronic kidney disease (CKD) (estimated glomerular filtration rate [eGFR] < 60 mL/min/1.73 m^2^ or urine albumin-to-creatinine ratio ≥ 30 mg/g).

### Statistical Analysis

The characteristics of the study population were compared between the group of MASLD and non-MASLD and the group of liver fibrosis and non-liver fibrosis. The chi-square test and *t*-test were applied for categorical variables and continuous variables, respectively. Univariate and multivariate logistic regression were conducted to investigate the associations of mEASIX with MASLD and liver fibrosis. Two models are structured for this analysis. Model 1 was adjusted for age, gender, race, and education level. Model 2 was adjusted for age, gender, race, education level, smoking, drinking, physical activity, total energy intake, vitamin D, sodium intake, overweight, diabetes, hypertension, dyslipidemia, CVDs, CKD, AST, ALT, albumin, and GGT in the outcome of MASLD, and adjusted for age, gender, education level, smoking, drinking, physical activity, total energy intake, vitamin D intake, sodium intake, overweight, diabetes, hypertension, dyslipidemia, CVDs, CKD, AST, ALT, albumin, and GGT in the outcome of liver fibrosis. These covariables were determined if they were significantly associated with the corresponding outcome using the univariate logistic regression analyses. In addition, the RCS was employed to detect possible nonlinear associations and filter for an optimal mEASIX cutoff in identifying MASLD and liver fibrosis. Furthermore, the interaction of magnesium intake with mEASIX in the association with MASLD/liver fibrosis was tested, and stratified analyses were conducted. To evaluate the joint associations of mEASIX and magnesium intake on MASLD/liver fibrosis, participants were divided into 4 groups based on the RCS cutoff of mEASIX and the median of magnesium intake. Finally, the diagnostic value of mEASIX for MASLD and liver fibrosis was described by receiver operating characteristic (ROC) curve. Weight variables were used in the analyses. A two-tailed *P < *.05 indicated statistical significance. Analyses were conducted using SAS 9.4 (SAS Institute Inc.; Cary, NC, USA). Python 3.9 (Python Software Foundation; Wilmington, DE, USA) was used for cleaning the data and drawing the RCS.

## Results

### Characteristics of the Study Population

The weighted prevalence of MASLD and liver fibrosis was 41.40% and 9.08%, respectively. The mEASIX was significantly higher in the MASLD and liver fibrosis group compared with the non-MASLD and non-liver fibrosis groups. The characteristics of the study population grouped by the outcomes (MASLD and liver fibrosis) are presented and compared in Table 1.

### Associations of Modified Endothelial Activation and Stress Index with Metabolic Dysfunction–Associated Steatotic Liver Disease and Liver Fibrosis

**Figure 2.**
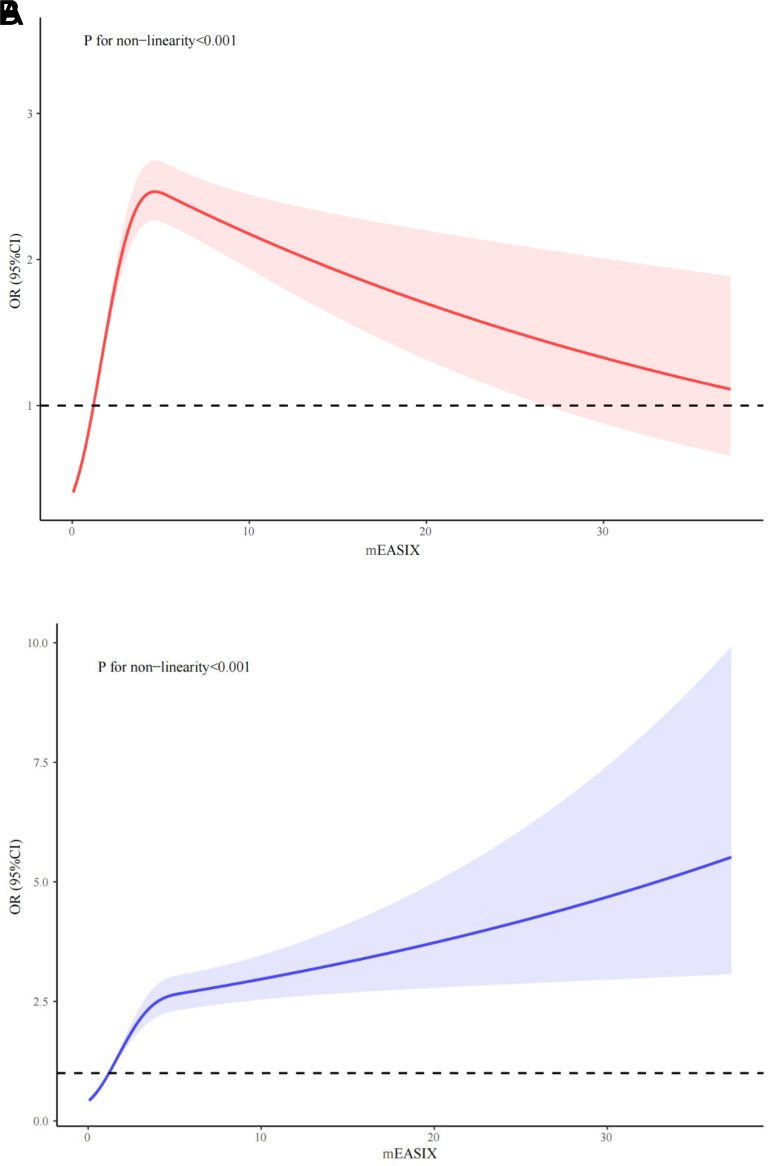
RCS for the associations of mEASIX with (A) MASLD and (B) liver fibrosis.

The RCS suggests nonlinear associations of mEASIX with MASLD and liver fibrosis (both *P*_ for non-linearity _< .001) (Figure 2). As mEASIX increases, the effect size (*β*) on MASLD grew steeply and then showed a steady and slightly downward trend (Figure 2A). However, the curve between mEASIX and liver fibrosis showed a persistent growth trend even though the growth rate slowed down as mEASIX increased (Figure 2B).

Modified endothelial activation and stress index was positively correlated with MASLD. Compared with the Q1 group, the odds of MASLD were significantly higher among those in Q2 (odds ratio [OR] = 1.62), Q3 (OR = 2.38), and Q4 (OR = 2.70) in the fully adjusted model. Participants with mEASIX ≥ 1.2 (RCS cutoff) also showed higher odds for MASLD (Table 2). Similarly, mEASIX was positively correlated with liver fibrosis. Compared with the Q1 group, the odds of liver fibrosis were significantly higher among those in Q3 (OR = 1.84) and Q4 (OR = 2.73) in the fully adjusted model. Participants with mEASIX ≥ 1.2 (RCS cutoff) also showed higher odds for liver fibrosis (Table 2).

### Diagnostic Value of Modified Endothelial Activation and Stress Index for Metabolic Dysfunction–Associated Steatotic Liver Disease and Liver Fibrosis

**Figure 3.**
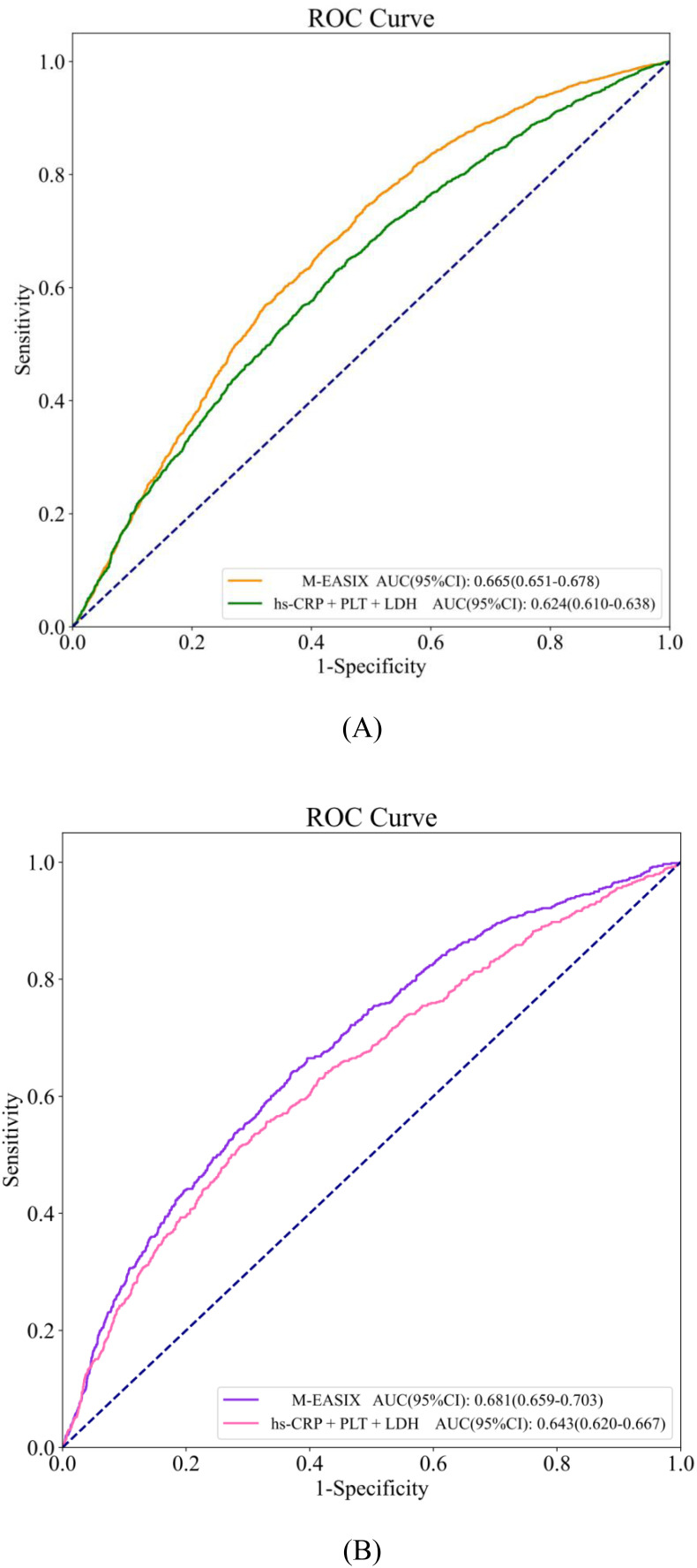
ROC curves of mEASIX with (A) MASLD and (B) liver fibrosis.

The diagnostic value of mEASIX was higher compared to the parameters used (MASLD: area under the curve (AUC) of mEASIX was 0.665, AUC of hs-CRP + PLT + LDH was 0.624, *P*_Delong test_ < .001. Liver fibrosis: AUC of mEASIX was 0.681, AUC of hs-CRP + PLT + LDH was 0.643, *P*_Delong test _< .001) (Figure 3 and Supplementary Table 1).

### Modification Effect of Magnesium Intake on the Association of Modified Endothelial Activation and Stress Index with Metabolic Dysfunction–Associated Steatotic Liver Disease and Liver Fibrosis

There was a significant interaction of the magnesium intake and the mEASIX level in the association with MASLD (*P*_ for interaction_ = .005) but not in the association with liver fibrosis (*P*_ for interaction_ = .364). When stratified according to the magnesium intake, the odds of MASLD and liver fibrosis for participants with high mEASIX were further elevated in the subgroup of low magnesium intake (MASLD: low Mg intake: 2.28; high Mg intake: 1.54; liver fibrosis: low Mg intake: 1.92; high Mg intake: 1.68; all *P* < .05) (Table 3).

### Combined Effect of Modified Endothelial Activation and Stress Index and Magnesium Intake on Metabolic Dysfunction–Associated Steatotic Liver Disease and Liver Fibrosis

The participants were grouped into 4 as shown in Table 4. Participants with high mEASIX and low Mg intake showed the highest odds of MASLD and liver fibrosis (both *P*_ for trend_ < .05) (Table 4).

### Sensitivity Analysis

Similar results were found based on the average magnesium intake for 2 nonconsecutive days (Supplementary Table 2).

## Discussion

The present study demonastrated nonlinear associations of mEASIX with MASLD and liver fibrosis. Participants with higher mEASIX had increased risk for MASLD and liver fibrosis. There was a significant interaction of the magnesium intake and the mEASIX level for the risk of MASLD. The risk for MASLD and liver fibrosis in patients with high mEASIX was more pronounced among participants with low magnesium intake compared to those with high magnesium intake.

Previous case-control studies with small sample sizes have demonstrated that patients with nonalcoholic fatty liver disease have significantly lower flow-mediated dilation (indicative of endothelial dysfunction),^28^ which is consistent with the positive association between mEASIX and MASLD in the current study. Nevertheless, limited studies have reported the status of endothelial dysfunction among liver fibrosis/cirrhosis with contradictory conclusions, mainly due to small samples and the complicated and expensive methods.^29^ Based on a relatively large representative sample, additional evidence was provided on the adverse effect of endothelial dysfunction on liver fibrosis. Furthermore, among patients with acute-on-chronic liver failure in the intensive care unit, EASIX showed high diagnostic potential to exclude clinically apparent infections and acceptable accuracy for predicting the requirement of hemodialysis and short-term mortality.^8^ Last but not least, elevated EASIX has been shown to be associated with the development and mortality of cardiometabolic diseases,^10^^,^^11^^,^^13^^,^^14^ which share pathological mechanisms with MASLD and liver fibrosis. The nonlinear associations between mEASIX and MASLD/liver fibrosis suggest that different mEASIX values may have disproportionately high risks. Similar nonlinear relationships between EASIX and stroke as well as cardiovascular mortality in patients with diabetes have been reported in previous studies.^11^^,^^13^ Additionally, concerning the modification effect of magnesium, evidence indicated that magnesium status can modify the oxidative stress/inflammation-related risk for the development of certain diseases. Elevated magnesium depletion status can increase the mortality related to hyperuricemia in coronary heart disease patients.^30^ Low magnesium intake may enhance the inverse association between CRP and fat-free mass.^31^

Several mechanisms may explain the observed relationships and the modification effect of magnesium intake. First, in the development of MASLD and liver fibrosis, changes in liver sinusoidal endothelial cells (LSECs) decrease hepatic blood flow, resulting in impaired hepatic microcirculation,^4^ followed by the elevated expression of cytokines and vascular adhesion molecules.^5^^,^^32^^,^^33^ Magnesium deficiency aggravates the production of these proinflammatory molecules such as tumor necrosis factor *α* (TNF-α), interleukin 1 (IL-1), IL-6, and vascular cell adhesion molecule-1 (VCAM),^34^ whereas high magnesium intake is negatively related to these markers.^35^^,^^36^ Second, increased oxidative stress in LSECs has been associated with microvascular blood flow restriction and endothelial autophagy impairment.^4^^,^^37^ Magnesium deficiency can exaggerate the oxidative stress by stimulating catecholamine release and the renin-angiotensin system, meanwhile decreasing expression and activity of the antioxidant enzymes such as glutathione peroxidase, catalase, and superoxide dismutase.^20^^,^^21^ Finally, aged LSECs may also generate the extracellular matrix and fibronectin, which promote liver fibrosis.^38^ Magnesium modulates the matrix metalloproteinase activity and the collagen and elastin turnover and helps to prevent calcium deposition.^39^

In terms of clinical implications, the current study reveals the potential of mEASIX as an indicator for early identification of MASLD or liver fibrosis. Based on routine clinical biomarkers such as LDH, PLTs, and hs-CRP, mEASIX provides a simple and cost-efficient tool for screening, especially in primary health-care institutions and community hospitals. The mEASIX with a threshold of 1.2 can be considered in the risk stratification system. Asymptomatic individuals with higher mEASIX (≥1.2) may benefit from earlier detection and intervention. However, more clinical validations are warranted before using it for risk stratification in different application scenarios. In addition, the current research highlighted the importance of taking measures to ensure adequate magnesium intake, such as increasing the consumption of magnesium-rich foods or choosing supplements.

The current study has several strengths. First, to the best of our knowledge, this is the first study examining the relationship between mEASIX and MASLD and liver fibrosis, which further emphasizes the role of endothelial dysfunction in the development mechanism of chronic liver disease based on a large sample size. Meanwhile, the current study provided a possible solution for the prevention of MASLD/liver fibrosis related to endothelial dysfunction as magnesium intake can be modified. Second, the noninvasive ultrasound transient elastography technique was applied to measure the extent of hepatic steatosis and liver stiffness with more accuracy. Third, the multi-stage stratified sampling method makes the findings generalizable to the entire US population. Despite these strengths, the current study has certain limitations. First, the cross-sectional design is not able to establish causality, which leaves the possibility of reverse causation. Prospective cohorts are warranted to further validate the association between mEASIX and MASLD/liver fibrosis, and clinical trials are needed to confirm the effect of magnesium consumption. Second, the current study is conducted only among US participants, and further studies in multiple populations are essential to generalize these findings. Third, a single 24-hour dietary recall may not adequately represent an individual’s long-term typical intake, particularly when dietary habits vary over time. Further research employing objective biomarkers (such as serum or erythrocyte magnesium levels) and their changes are warranted to further verify these findings. Finally, although the current study controlled many conventional variables, the potential effects of residual confounding factors, such as phytic acid and medications that disturb magnesium levels (diuretics and proton pump inhibitors), cannot be ruled out.

In conclusion, the current study demonstrated positive nonlinear associations of mEASIX with MASLD and liver fibrosis. Low magnesium intake may exacerbate endothelial dysfunction-related risk for MASLD and liver fibrosis.

## Figures and Tables

**Table 1.** Characteristics of the Study Population

**Variables**	**Total (n = 5960)**	**Non-MASLD (n = 3421)**	**MASLD (n = 2539)**	*P*	**Non-Liver Fibrosis (n = 5324)**	**Liver Fibrosis (n = 636)**	*P*
mEASIX, mean (SE)	2.33 (0.10)	1.86 (0.11)	3.01 (0.16)	<.001	2.11 (0.09)	4.53 (0.58)	<.001
mEASIX category, n (%)				<.001			<.001
Low (< 1.2)	2972 (52.46)	2055 (62.92)	917 (37.65)		2798 (54.66)	174 (30.44)	
High (≥ 1.2)	2988 (47.54)	1366 (37.08)	1622 (62.35)		2526 (45.34)	462 (69.56)	
mEASIX quartiles, n (%)				<.001			<.001
Q1	1397 (24.99)	1103 (34.21)	294 (11.94)		1341 (26.49)	56 (9.96)	
Q2	1402 (24.98)	862 (26.60)	540 (22.69)		1301 (25.83)	101 (16.41)	
Q3	1529 (24.98)	766 (20.63)	763 (31.13)		1365 (24.75)	164 (27.25)	
Q4	1632 (25.05)	690 (18.56)	942 (34.24)		1317 (22.92)	315 (46.37)	
Magnesium intake, mg/d, Mean (SE)	327.52 (5.05)	324.96 (4.43)	331.15 (9.50)	.533	328.64 (4.91)	316.30 (14.55)	.382
Magnesium intake, n (%)				.779			.337
≥ 286.74 mg/d	2751 (50.25)	1563 (49.99)	1188 (50.62)		2478 (50.56)	273 (47.18)	
< 286.74 mg/d	3209 (49.75)	1858 (50.01)	1351 (49.38)		2846 (49.44)	363 (52.82)	
mEASIX × Mg intake group, n (%)				<.001			<.001
Low mEASIX and high Mg intake	1530 (29.49)	1004 (33.65)	526 (23.60)		1443 (30.64)	87 (17.97)	
Low mEASIX and low Mg intake	1442 (22.97)	1051 (29.26)	391 (14.05)		1355 (24.01)	87 (12.47)	
High mEASIX and high Mg intake	1221 (20.76)	559 (16.34)	662 (27.02)		1035 (19.92)	186 (29.20)	
High mEASIX and low Mg intake	1767 (26.78)	807 (20.74)	960 (35.33)		1491 (25.43)	276 (40.35)	
Age, years, mean (SE)	46.76 (0.61)	44.39 (0.65)	50.12 (0.70)	<.001	46.25 (0.59)	51.89 (1.15)	<.001
Gender, n (%)				<.001			.011
Male	2678 (45.51)	1418 (40.40)	1260 (52.74)		2351 (44.69)	327 (53.71)	
Female	3282 (54.49)	2003 (59.60)	1279 (47.26)		2973 (55.31)	309 (46.29)	
Race, n (%)				<.001			.417
Non-Hispanic White	2114 (62.19)	1185 (62.94)	929 (61.11)		1874 (62.10)	240 (63.07)	
Non-Hispanic Black	1497 (10.85)	958 (12.19)	539 (8.95)		1320 (10.66)	177 (12.80)	
Others	2349 (26.96)	1278 (24.86)	1071 (29.93)		2130 (27.25)	219 (24.13)	
PIR, n (%)				.382			.379
< 1.3	1646 (18.99)	979 (19.42)	667 (18.37)		1488 (19.16)	158 (17.26)	
≥ 1.3	4314 (81.01)	2442 (80.58)	1872 (81.63)		3836 (80.84)	478 (82.74)	
Education level, n (%)				.010			.008
High school and below	2361 (36.03)	1303 (34.13)	1058 (38.72)		2077 (35.12)	284 (45.20)	
College and above	3599 (63.97)	2118 (65.87)	1481 (61.28)		3247 (64.88)	352 (54.80)	
Smoking, n (%)				.015			.083
No	3872 (63.59)	2303 (65.21)	1569 (61.29)		3494 (64.00)	378 (59.52)	
Yes	2088 (36.41)	1118 (34.79)	970 (38.71)		1830 (36.00)	258 (40.48)	
Alcohol, n (%)				.428			.034
No	1696 (21.14)	945 (20.54)	751 (21.98)		1492 (20.70)	204 (25.52)	
Yes	4264 (78.86)	2476 (79.46)	1788 (78.02)		3832 (79.30)	432 (74.48)	
Physical activity, n (%)				<.001			<.001
< 450 MET·min/week	517 (7.91)	276 (6.56)	241 (9.83)		452 (7.42)	65 (12.79)	
≥ 450 MET·min/week	4062 (73.34)	2444 (77.24)	1618 (67.83)		3673 (74.58)	389 (60.95)	
Unknown	1381 (18.75)	701 (16.21)	680 (22.35)		1199 (18.00)	182 (26.26)	
Total energy intake, kcal, Mean (SE)	2098.27 (16.80)	2039.67 (21.67)	2181.21 (27.42)	<.001	2088.29 (17.21)	2198.28 (57.82)	.072
Vitamin D intake, mcg, Mean (SE)	21.11 (1.38)	19.77 (1.88)	22.99 (2.29)	.315	21.06 (1.45)	21.58 (2.49)	.846
Sodium intake, mg, mean (SE)	3415.73 (34.53)	3313.48 (43.62)	3560.46 (45.88)	<.001	3402.48 (36.42)	3548.46 (97.96)	.167
BMI, kg/m^2^, mean (SE)	29.61 (0.19)	26.65 (0.18)	33.80 (0.25)	<.001	28.90 (0.18)	36.74 (0.61)	<.001
Overweight, n (%)				<.001			<.001
No	1550 (26.66)	1380 (41.54)	170 (5.60)		1486 (28.08)	64 (12.43)	
Yes	4410 (73.34)	2041 (58.46)	2369 (94.40)		3838 (71.92)	572 (87.57)	
Diabetes, n (%)				<.001			<.001
No	4803 (85.58)	3052 (92.95)	1751 (75.15)		4451 (87.90)	352 (62.29)	
Yes	1157 (14.42)	369 (7.05)	788 (24.85)		873 (12.10)	284 (37.71)	
Hypertension, n (%)				<.001			<.001
No	3265 (60.66)	2190 (71.45)	1075 (45.39)		3069 (63.01)	196 (37.11)	
Yes	2695 (39.34)	1231 (28.55)	1464 (54.61)		2255 (36.99)	440 (62.89)	
Dyslipidemia, n (%)				<.001			<.001
No	2079 (35.40)	1517 (45.74)	562 (20.77)		1919 (36.50)	160 (24.45)	
Yes	3881 (64.60)	1904 (54.26)	1977 (79.23)		3405 (63.50)	476 (75.55)	
CVDs, n (%)				<.001			<.001
No	4712 (82.21)	2848 (86.75)	1864 (75.77)		4301 (83.50)	411 (69.25)	
Yes	1248 (17.79)	573 (13.25)	675 (24.23)		1023 (16.50)	225 (30.75)	
CKD, n (%)				<.001			<.001
No	5044 (87.89)	2964 (89.69)	2080 (85.33)		4584 (89.11)	460 (75.69)	
Yes	916 (12.11)	457 (10.31)	459 (14.67)		740 (10.89)	176 (24.31)	
AST, U/L, mean (SE)	21.33 (0.25)	20.31 (0.31)	22.77 (0.38)	<.001	20.72 (0.23)	27.36 (1.11)	<.001
ALT, U/L, mean (SE)	22.07 (0.31)	18.74 (0.35)	26.77 (0.56)	<.001	21.27 (0.30)	30.07 (1.31)	<.001
Albumin, g/dL, mean (SE)	4.13 (0.01)	4.16 (0.01)	4.09 (0.01)	<.001	4.14 (0.01)	4.04 (0.02)	<.001
GGT, IU/L, mean (SE)	26.89 (0.46)	22.09 (0.63)	33.69 (0.73)	<.001	24.91 (0.47)	46.71 (3.06)	<.001

Mean ± standard error (SE) for continuous variables; number (proportion) for categorical variables. *P* was obtained from *t*-test for continuous variables and the chi-square test for categorical variables.

CKD, chronic kidney disease; CVDs, cardiovascular diseases; GGT, gamma glutamyl transferase; MASLD, metabolic dysfunction–associated steatotic liver disease; mEASIX, modified endothelial activation and stress index; MET.min, metabolic equivalent · minutes; PIR, poverty income ratio; SE, standard error.

**Table 2.** Association Between Modified Endothelial Activation and Stress Index and Metabolic Dysfunction–Associated Steatotic Liver Disease and Liver Fibrosis

	Model 1		Model 2	
	OR (95% CI)	*P*	OR (95% CI)	*P*
**MASLD**				
mEASIX	1.08 (1.03-1.12)	<.001	1.02 (1.01-1.05)	.015
**mEASIX quartiles**				
Q1	Reference		Reference	
Q2	2.31 (1.59-3.36)	<.001	1.62 (1.06-2.48)	.028
Q3	4.15 (3.02-5.70)	<.001	2.38 (1.65-3.43)	<.001
Q4	5.51 (3.80-8.00)	<.001	2.70 (1.72-4.24)	<.001
**RCS cutoff**				
< 1.2	Reference		Reference	
≥ 1.2	2.91 (2.32-3.67)	<.001	1.84 (1.39-2.43)	<.001
**Liver fibrosis**				
mEASIX	1.06 (1.03-1.08)	<.001	1.03 (1.01-1.04)	<.001
**mEASIX quartiles**				
Q1	Reference		Reference	
Q2	1.55 (0.72-3.34)	.253	1.31 (0.61-2.81)	.472
Q3	2.67 (1.57-4.54)	<.001	1.85 (1.05-3.25)	.033
Q4	5.09 (3.25-7.98)	<.001	2.77 (1.67-4.58)	<.001
**RCS cutoff**				
< 1.2	Reference		Reference	
≥ 1.2	2.69 (2.14-3.38)	<.001	1.75 (1.31-2.32)	<.001

Model 1: adjusted for age, gender, race, education level.

Model 2:

MASLD: adjusted for age, gender, race, education level, smoking, drinking, physical activity, total energy intake, vitamin D intake, sodium intake, overweight, diabetes, hypertension, dyslipidemia, cardiovascular diseases, chronic kidney disease, AST, ALT, albumin, and gamma glutamyl transferase.

Liver fibrosis: adjusted for age, gender, education level, smoking, drinking, physical activity, total energy intake, vitamin D intake, sodium intake, overweight, diabetes, hypertension, dyslipidemia, cardiovascular diseases, chronic kidney disease, AST, ALT, albumin, and gamma glutamyl transferase.

MASLD, metabolic dysfunction–associated steatotic liver disease; mEASIX, modified endothelial activation and stress index; OR, odds ratio; RCS, restricted cubic spline.

**Table 3.** Associations of Modified Endothelial Activation and Stress Index with Metabolic Dysfunction–Associated Steatotic Liver Disease and Liver Fibrosis in Magnesium Intake Subgroups

	High Mg Intake (n = 2751)		Low Mg Intake (n = 3209)	
	OR (95% CI)	*P*	OR (95% CI)	*P*
MASLD				
mEASIX < 1.2	Reference		Reference	
mEASIX ≥ 1.2	1.54 (1.13-2.10)	.008	2.28 (1.63-3.20)	<.001
Liver fibrosis				
mEASIX < 1.2	Reference		Reference	
mEASIX ≥ 1.2	1.65 (1.08-2.52)	.023	1.95 (1.42-2.68)	<.001

MASLD: adjusted for age, gender, race, education level, smoking, drinking, physical activity, total energy intake, vitamin D intake, sodium intake, overweight, diabetes, hypertension, dyslipidemia, cardiovascular diseases, chronic kidney disease, AST, ALT, albumin, and gamma glutamyl transferase.

Liver fibrosis: adjusted for age, gender, education level, smoking, drinking, physical activity, total energy intake, vitamin D intake, sodium intake, overweight, diabetes, hypertension, dyslipidemia, cardiovascular diseases, chronic kidney disease, AST, ALT, albumin, and gamma glutamyl transferase.

MASLD, metabolic dysfunction–associated steatotic liver disease; mEASIX, modified endothelial activation and stress index; Mg, magnesium; OR, odds ratio.

**Table 4.** Joint Associations of Modified Endothelial Activation and Stress Index and Magnesium Intake on Metabolic Dysfunction–Associated Steatotic Liver Disease and Liver Fibrosis

	OR (95% CI)	*P*	*P* for Trend
MASLD			
Low mEASIX and high Mg intake	Reference		<.001
Low mEASIX and low Mg intake	0.90 (0.63-1.30)	.574	
High mEASIX and high Mg intake	1.47 (1.06-2.02)	.022	
High mEASIX and low Mg intake	2.06 (1.36-3.14)	.001	
Liver fibrosis			
Low mEASIX and high Mg intake	Reference		<.001
Low mEASIX and low Mg intake	1.01 (0.60-1.70)	.959	
High mEASIX and high Mg intake	1.54 (1.01-2.35)	.047	
High mEASIX and low Mg intake	1.98 (1.22-3.22)	.007	

MASLD: adjusted for age, gender, race, education level, smoking, drinking, physical activity, total energy intake, vitamin D intake, sodium intake, overweight, diabetes, hypertension, dyslipidemia, cardiovascular diseases, chronic kidney disease, AST, ALT, albumin, and gamma glutamyl transferase.

Liver fibrosis: adjusted for age, gender, education level, smoking, drinking, physical activity, total energy intake, vitamin D intake, sodium intake, overweight, diabetes, hypertension, dyslipidemia, cardiovascular diseases, chronic kidney disease, AST, ALT, albumin, and gamma glutamyl transferase.

MASLD, metabolic dysfunction–associated steatotic liver disease; mEASIX, modified endothelial activation and stress index; Mg, magnesium; OR, odds ratio.

**Supplementary Table 1.** Diagnostic value of mEASIX on MASLD and liver fibrosis

	AUC (95% CI)	Delong Test *Z*	*P*
MASLD			
mEASIX	0.665(0.651-0.678)	7.72	<.001
hs-CRP+PLT+LDH	0.624(0.610-0.638)		
Liver fibrosis			
mEASIX	0.681(0.659-0.703)	3.79	<.001
hs-CRP+PLT+LDH	0.643(0.620-0.667)		

**Supplementary Table 2.** Associations of mEASIX with MASLD and liver fibrosis in magnesium intake subgroups (average magnesium intake based on 2 nonconsecutive days)

	High Mg intake (n = 2319)		Low Mg intake (n = 2816)	
	OR (95% CI)	*P*	OR (95% CI)	*P*
MASLD				
mEASIX < 1.2	Reference		Reference	
mEASIX ≥ 1.2	1.74 (1.09-2.79)	.022	2.00 (1.37-2.91)	<.001
Liver fibrosis				
mEASIX < 1.2	Reference		Reference	
mEASIX ≥ 1.2	1.63 (0.97-2.74)	.065	1.85 (1.15-2.99)	.013

mEASIX: modified endothelial activation and stress index; MASLD: metabolic dysfunction-associated steatotic liver disease; OR: odds ratio; CI: confidence interval.

MASLD: adjusted for age, gender, race, education level, smoking, drink, physical activity, total energy intake, vitamin D intake, sodium intake, overweight, diabetes, hypertension, dyslipidemia, CVDs, CKD, AST, ALT, albumin, and GGT

Liver fibrosis: adjusted for age, gender, education level, smoking, drink, physical activity, total energy intake, vitamin D intake, sodium intake, overweight, diabetes, hypertension, dyslipidemia, CVDs, CKD, AST, ALT, albumin, and GGT

## Data Availability

The data that support the findings of the current study are available on request from the corresponding author.
